# *Candida albicans* Is Resistant to Polyglutamine Aggregation and Toxicity

**DOI:** 10.1534/g3.116.035675

**Published:** 2016-11-01

**Authors:** Michelle D. Leach, TaeHyung Kim, Sonja E. DiGregorio, Cathy Collins, Zhaolei Zhang, Martin L. Duennwald, Leah E. Cowen

**Affiliations:** * Aberdeen Fungal Group, University of Aberdeen, Institute of Medical Sciences, Foresterhill, AB25 2ZD, UK; † Department of Molecular Genetics, University of Toronto, Ontario M5S 1A8, Canada; ‡ Banting and Best Department of Medical Research, The Donnelly Centre, University of Toronto, Ontario, M5S 3E1, Canada; § Department of Computer Science, University of Toronto, Ontario M5S 2E4, Canada; ** Department of Pathology, Schulich School of Medicine and Dentistry, University of Western Ontario, London, Ontario N6A 5C1, Canada

**Keywords:** *Candida albicans*, PolyQ, Aggregation, Toxicity, *Saccharomyces cerevisiae*

## Abstract

Disruption of protein quality control can be detrimental, having toxic effects on single cell organisms and contributing to neurodegenerative diseases such as Alzheimer’s, Parkinson’s and Huntington’s in humans. Here, we examined the effects of polyglutamine (polyQ) aggregation in a major fungal pathogen of humans, *Candida albicans*, with the goal of identifying new approaches to disable this fungus. However, we discovered that expression of polyQ stretches up to 230Q had no effect on *C. albicans* ability to grow and withstand proteotoxic stress. Bioinformatics analysis demonstrates that *C. albicans* has a similarly glutamine-rich proteome to the unicellular fungus *Saccharomyces cerevisiae*, which exhibits polyQ toxicity with as few as 72Q. Surprisingly, global transcriptional profiles indicated no significant change upon induction of up to 230Q. Proteomic analysis highlighted two key interactors of 230Q, Sis1 and Sgt2; however, loss of either protein had no additional effect on *C. albicans* toxicity. Our data suggest that *C. albicans* has evolved powerful mechanisms to overcome the toxicity associated with aggregation-prone proteins, providing a unique model for studying polyQ-associated diseases.

All cells use elaborate strategies to maintain cellular protein homeostasis and cope with environmental challenges. During stress, protein homeostasis is often compromised, eliciting protein unfolding, misfolding, and aggregation. Major cellular pathways controlling proteostasis in eukaryotic cells include the heat shock response (HSR) and the unfolded protein response (UPR), which involve molecular chaperones that aid in proper protein folding and prevent protein misfolding (Frydman 2001; Leach and Cowen 2014b). Both the HSR and UPR are rapidly activated upon stress to restore homeostasis and thereby prevent the toxic consequences of protein misfolding. Numerous neurodegenerative diseases are characterized by the accumulation of toxic misfolded proteins and a malfunctioning protein quality control system (Ross and Poirier 2004; Thomas *et al.* 1995). For example, genetic mutations resulting in aberrant expansions of polyQ repeats cause nine neurodegenerative disorders, including Huntington’s disease and spinocerebellar ataxias (Fan *et al.* 2014). In all these diseases, CAG trinucleotide repeat expansions in the translated region of specific genes lead to polyQ length-dependent aggregation, toxicity, and eventually neurodegeneration (Duyao *et al.* 1993).

PolyQ expansion proteins misfold into β sheet-rich, aggregating structures (Wickner *et al.* 2007), which mediate abnormal interactions with other proteins via exposed hydrophobic surfaces, thus leading to functional impairment and cellular toxicity (Chiti and Dobson 2006; Bolognesi *et al.* 2010; Olzscha *et al.* 2011). The presence of coiled-coil domains in polyQ peptides, which regulate polyQ aggregation and insolubility, are thought to enhance the pathogenesis of polyQ expansion diseases (Fiumara *et al.* 2010). PolyQ aggregation is thought to interfere with cellular protein quality control, including protein degradation, molecular chaperone-mediated protein folding, and the activity of the HSR and the UPR, thereby propagating protein misfolding within cells and entire tissues with toxic consequences (Bence *et al.* 2001; Gidalevitz *et al.* 2006).

Much of our understanding of polyQ-mediated toxicity and its interactions with cellular protein quality control comes from experiments expressing amino-terminal fragments of huntingtin (Htt)-containing polyQ expansions in the yeast *Saccharomyces cerevisiae* (Duennwald *et al.* 2006a,b; Krobitsch and Lindquist 2000). While only a small fraction of human proteins contain polyQ domains (defined as more than 10 consecutive glutamines in a protein), the yeast proteome is comparatively Q-rich (Michelitsch and Weissman 2000), with more than 50 polyQ proteins (Krobitsch and Lindquist 2000). Another organism that harbors unusually high numbers of polyQ proteins is the social amoeba *Dictyostelium discoideum* (Malinovska *et al.* 2015), which has an exceptionally high level of resilience to polyQ aggregation and toxicity in the absence of cellular stress (Malinovska *et al.* 2015; Santarriaga *et al.* 2015). As a consequence, *D. discoideum* was thought to exemplify the evolution of robust protein quality control machinery to cope with a proteome enriched in aggregation-prone proteins.

We aimed to understand how the major fungal pathogen of humans, *Candida albicans*, responds to polyQ proteins. *C. albicans* is a natural commensal of the human mucosal microbiota; however, in immunocompromised patients it can disseminate, accounting for over 400,000 life threatening infections world-wide every year (Horn *et al.* 2009). With limited drugs available and antifungal drug resistance on the rise (Pfaller 2012), we aimed to explore cellular protein quality control in *C. albicans* and its potential as a new therapeutic target. To this end, we expressed polyQ expansion proteins in *C. albicans* and assessed polyQ aggregation and toxicity. Our experiments document that *C. albicans*, whose proteome has a similar polyQ content to that of *S. cerevisiae*, is remarkably resistant to both polyQ aggregation and toxicity. Indeed, expression of expanded polyQ regions up to 230Qs had no effect on global transcription, resistance to stress, or general fitness of *C. albicans* cells. Heat shock and other proteotoxic stress conditions induced weak polyQ aggregation, without any toxic consequences. Our results, together with studies in *S. cerevisiae*, *Schizosaccharomyces pombe*, *Drosophila melanogaster*, *Caenorhabditis elegans*, and other model organisms, reveal that the prevalence of aggregation-prone proteins in an organism’s proteome does not automatically determine the capacity of the accompanying cellular protein quality control machinery to modulate protein aggregation and toxicity.

## Materials and Methods

### Strains and growth conditions

All strains are listed in Table 1. *C. albicans* strains were grown in YPD (1% yeast extract, 2% bactopeptone, and 2% glucose) (Sherman 1991). To induce expression of Htt polyglutamine expansions, strains grown overnight in YPD at 30° were diluted to OD_600_ 0.2 in the absence or presence of 50 µg/ml doxycycline (BD Biosciences) for 24 hr. Cells were again diluted to OD_600_ 0.05 under the same conditions and grown to midlog phase at the temperature indicated (∼6 hr) prior to microscopy, RNA extraction, spotting assays, Western blots, or semidenaturating detergent agarose gel electrophoresis (SDD-AGE) (Halfmann and Lindquist 2008). *S. cerevisiae* strains were grown in selective media containing 2% glucose overnight before being diluted to OD_600_ 0.2 in selective media containing 2% glucose or 2% galactose to midlog phase (∼7 hr) to induce expression of the Htt polyglutamine expansions. A 30–42°, heat shock was imposed as described previously (Leach *et al.* 2012c). All drugs were added at the concentrations stated.

**Table 1:** C. *albicans* strains

Strain	Genotype	Source
SN95	*arg4*∆*/arg4*∆ *his1*∆*/his1*∆ *URA3/ura3*:: λ*imm434 IRO1/iro1*::λ*imm434*	Noble and Johnson (2005)
CaLC3067 *HSP104/HSP104*-GFP	*arg4*∆*/arg4*∆ *his1*∆*/his1*∆ *URA3/ura3*:: λ*imm434 IRO1/iro1*::λ*imm434 HSP104/HSP104-GFP*	This study
CaLC3069 25Q-1	*arg4*∆*/arg4*∆ *his1*∆*/his1*∆ *URA3/ura3*:: λ*imm434 IRO1/iro1*::λ*imm434 HSP104/HSP104-GFP*, *ADH1/adh1*::*Ptet-FLAG-25Q-RFP*	This study
CaLC3070 25Q-2	*arg4*∆*/arg4*∆ *his1*∆*/his1*∆ *URA3/ura3*:: λ*imm434 IRO1/iro1*::λ*imm434 HSP104/HSP104-GFP*, *ADH1/adh1*::*Ptet-FLAG-25Q-RFP*	This study
CaLC3069 72Q-1	*arg4*∆*/arg4*∆ *his1*∆*/his1*∆ *URA3/ura3*:: λ*imm434 IRO1/iro1*::λ*imm434 HSP104/HSP104-GFP*, *ADH1/adh1*::*Ptet-FLAG-72Q-RFP*	This study
CaLC3070 72Q-2	*arg4*∆*/arg4*∆ *his1*∆*/his1*∆ *URA3/ura3*:: λ*imm434 IRO1/iro1*::λ*imm434 HSP104/HSP104-GFP*, *ADH1/adh1*::*Ptet-FLAG-72Q-RFP*	This study
CaLC3252 103Q-1	*arg4*∆*/arg4*∆ *his1*∆*/his1*∆ *URA3/ura3*:: λ*imm434 IRO1/iro1*::λ*imm434 ADH1/adh1*::*Ptet-FLAG-103Q-RFP*	This study
CaLC3253 103Q-2	*arg4*∆*/arg4*∆ *his1*∆*/his1*∆ *URA3/ura3*:: λ*imm434 IRO1/iro1*::λ*imm434 ADH1/adh1*::*Ptet-FLAG-103Q-RFP*	This study
CaLC3256 230Q-1	*arg4*∆*/arg4*∆ *his1*∆*/his1*∆ *URA3/ura3*:: λ*imm434 IRO1/iro1*::λ*imm434 ADH1/adh1*::*Ptet-FLAG-230Q-RFP*	This study
CaLC3257 230Q-2	*arg4*∆*/arg4*∆ *his1*∆*/his1*∆ *URA3/ura3*:: λ*imm434 IRO1/iro1*::λ*imm434 ADH1/adh1*::*Ptet-FLAG-230Q-RFP*	This study
W303a	*a can1-100*, *his3-11,15*, *leu2-3,112*, *trp1-1*, *ura3-1*, *ade2-1*	Duennwald *et al.* (2006b)
25Q	*As W303a GAL-FLAG-25Q-CFP*	Duennwald *et al.* (2006b)
72Q	*As W303a GAL-FLAG-72Q-CFP*	Duennwald *et al.* (2006b)
103Q *rnq*Δ	*As W303a GAL-FLAG-103Q-CFP rnq*Δ	Duennwald *et al.* (2006b)
103Q *RNQ*	*As W303a GAL-FLAG-103Q-CFP RNQ*	Duennwald *et al.* (2006b)
CaLC4012 *hsp104*Δ/*hsp104*Δ	*arg4*∆*/arg4*∆ *his1*∆*/his1*∆ *URA3/ura3*:: λ*imm434 IRO1/iro1*::λ*imm434 hsp104*Δ/*hsp104*Δ::*FRT*	This study
CaLC4088 *hsp104*Δ/*hsp104*Δ +103Q-1	*arg4*∆*/arg4*∆ *his1*∆*/his1*∆ *URA3/ura3*:: λ*imm434 IRO1/iro1*::λ*imm434 hsp104*Δ/*hsp104*Δ::*FRT*, *ADH1/adh1*::*Ptet-FLAG-103Q-RFP*	This study
CaLC4082 *hsp104*Δ/*hsp104*Δ +103Q-2	*arg4*∆*/arg4*∆ *his1*∆*/his1*∆ *URA3/ura3*:: λ*imm434 IRO1/iro1*::λ*imm434 hsp104*Δ/*hsp104*Δ::*FRT*, *ADH1/adh1*::*Ptet-FLAG-103Q-RFP*	This study
CaLC4083 *hsp104*Δ/*hsp104*Δ +230Q-1	*arg4*∆*/arg4*∆ *his1*∆*/his1*∆ *URA3/ura3*:: λ*imm434 IRO1/iro1*::λ*imm434 hsp104*Δ/*hsp104*Δ::*FRT*, *ADH1/adh1*::*Ptet-FLAG-230Q-RFP*	This study
CaLC4084 *hsp104*Δ/*hsp104*Δ +230Q-2	*arg4*∆*/arg4*∆ *his1*∆*/his1*∆ *URA3/ura3*:: λ*imm434 IRO1/iro1*::λ*imm434 hsp104*Δ/*hsp104*Δ::*FRT*, *ADH1/adh1*::*Ptet-FLAG-230Q-RFP*	This study
CaLC4385 *sgt2*Δ/*sgt2*Δ	*arg4*∆*/arg4*∆ *his1*∆*/his1*∆ *URA3/ura3*::*imm434 IRO1/iro1*::*imm434 sgt2*Δ/*sgt2*Δ::*FRT*	This study
CaLC4397 *sgt2*Δ/*sgt2*Δ+103Q-1	*arg4*∆*/arg4*∆ *his1*∆*/his1*∆ *URA3/ura3*::*imm434 IRO1/iro1*::*imm434 sgt2*Δ/*sgt2*Δ::*FRT*, *ADH1/adh1*::*Ptet-FLAG-103Q-RFP*	This study
CaLC4398 *sgt2*Δ/*sgt2*Δ+103Q-2	*arg4*∆*/arg4*∆ *his1*∆*/his1*∆ *URA3/ura3*::*imm434 IRO1/iro1*::*imm434 sgt2*Δ/*sgt2*Δ::*FRT*, *ADH1/adh1*::*Ptet-FLAG-103Q-RFP*	This study
CaLC4399 *sgt2*Δ/*sgt2*Δ+230Q-1	*arg4*∆*/arg4*∆ *his1*∆*/his1*∆ *URA3/ura3*::*imm434 IRO1/iro1*::*imm434 sgt2*Δ/*sgt2*Δ::*FRT*, *ADH1/adh1*::*Ptet-FLAG-230Q-RFP*	This study
CaLC4400 *sgt2*Δ/*sgt2*Δ+230Q-2	*arg4*∆*/arg4*∆ *his1*∆*/his1*∆ *URA3/ura3*::*imm434 IRO1/iro1*::*imm434 sgt2*Δ/*sgt2*Δ::*FRT*, *ADH1/adh1*::*Ptet-FLAG-230Q-RFP*	This study
CaLC4450 *sis1*Δ/*sis1*Δ	*arg4*∆*/arg4*∆ *his1*∆*/his1*∆ *URA3/ura3*::*imm434 IRO1/iro1*::*imm434 sis1*Δ/*sis1*Δ::*FRT*	This study
CaLC4462 *sis1*Δ/*sis1*Δ+103Q-1	*arg4*∆*/arg4*∆ *his1*∆*/his1*∆ *URA3/ura3*::*imm434 IRO1/iro1*::*imm434 sis1*Δ/*sis1*Δ::*FRT*, *ADH1/adh1*::*Ptet-FLAG-103Q-RFP*	This study
CaLC4463 *sis1*Δ/*sis1*Δ+103Q-2	*arg4*∆*/arg4*∆ *his1*∆*/his1*∆ *URA3/ura3*::*imm434 IRO1/iro1*::*imm434 sis1*Δ/*sis1*Δ::*FRT*, *ADH1/adh1*::*Ptet-FLAG-103Q-RFP*	This study
CaLC4464 *sis1*Δ/*sis1*Δ+230Q-1	*arg4*∆*/arg4*∆ *his1*∆*/his1*∆ *URA3/ura3*::*imm434 IRO1/iro1*::*imm434 sis1*Δ/*sis1*Δ::*FRT*, *ADH1/adh1*::*Ptet-FLAG-230Q-RFP*	This study
CaLC4465 *sis1*Δ/*sis1*Δ+230Q-2	*arg4*∆*/arg4*∆ *his1*∆*/his1*∆ *URA3/ura3*::*imm434 IRO1/iro1*::*imm434 sis1*Δ/*sis1*Δ::*FRT*, *ADH1/adh1*::*Ptet-FLAG-230Q-RFP*	This study
CaLC3491 BWP17 + CIp30	*ura3*Δ::*imm434/ura3*Δ::*imm434 arg4*Δ::*hisG/arg4*Δ::*hisG his1*Δ::*hisG/his1*Δ::*hisG*, *CIp30 (ARG4*, *HIS1*, *URA3)*	This study
CaLC4483 BWP17+103Q-1	*ura3*Δ::*imm434/ura3*Δ::*imm434 arg4*Δ::*hisG/arg4*Δ::*hisG his1*Δ::*hisG/his1*Δ::*hisG*, *CIp30 (ARG4*, *HIS1*, *URA3)*, *ADH1/adh1*::*Ptet-FLAG-103Q-RFP*	This study
CaLC4484 BWP17+103Q-2	*ura3*Δ::*imm434/ura3*Δ::*imm434 arg4*Δ::*hisG/arg4*Δ::*hisG his1*Δ::*hisG/his1*Δ::*hisG*, *CIp30 (ARG4*, *HIS1*, *URA3)*, *ADH1/adh1*::*Ptet-FLAG-103Q-RFP*	This study
CaLC4485 BWP17+230Q-1	*ura3*Δ::*imm434/ura3*Δ::*imm434 arg4*Δ::*hisG/arg4*Δ::*hisG his1*Δ::*hisG/his1*Δ::*hisG*, *CIp30 (ARG4*, *HIS1*, *URA3)*, *ADH1/adh1*::*Ptet-FLAG-230Q-RFP*	This study
CaLC4486 BWP17+230Q-2	*ura3*Δ::*imm434/ura3*Δ::*imm434 arg4*Δ::*hisG/arg4*Δ::*hisG his1*Δ::*hisG/his1*Δ::*hisG*, *CIp30 (ARG4*, *HIS1*, *URA3)*, *ADH1/adh1*::*Ptet-FLAG-230Q-RFP*	This study
CaLC2369 *ubi4*Δ/*ubi4*Δ	*ura3*Δ::*imm434/ura3*Δ::*imm434 arg4*Δ::*hisG/arg4*Δ::*hisG his1*Δ::*hisG/his1*Δ::*hisG*, *UBI4/loxP-HIS1-loxP*::*ubi4*Δ*/loxP-URA-loxP*::*ubi4*Δ, *NRG1-3xHA-ARG4*	This study
CaLC4487 *ubi4*Δ/*ubi4*Δ +103Q-1	*ura3*Δ::*imm434/ura3*Δ::*imm434 arg4*Δ::*hisG/arg4*Δ::*hisG his1*Δ::*hisG/his1*Δ::*hisG*, *UBI4/loxP-HIS1-loxP*::*ubi4*Δ*/loxP-URA-loxP*::*ubi4*Δ, *NRG1-3xHA-ARG4*, *ADH1/adh1*::*Ptet-FLAG-103Q-RFP*	This study
CaLC4488 *ubi4*Δ/*ubi4*Δ +103Q-2	*ura3*Δ::*imm434/ura3*Δ::*imm434 arg4*Δ::*hisG/arg4*Δ::*hisG his1*Δ::*hisG/his1*Δ::*hisG*, *UBI4/loxP-HIS1-loxP*::*ubi4*Δ*/ loxP-URA-loxP*::*ubi4*Δ, *NRG1-3xHA-ARG4*, *ADH1/adh1*::*Ptet-FLAG-103Q-RFP*	This study
CaLC4489 *ubi4*Δ/*ubi4*Δ +230Q-1	*ura3*Δ::*imm434/ura3*Δ::*imm434 arg4*Δ::*hisG/arg4*Δ::*hisG his1*Δ::*hisG/his1*Δ::*hisG*, *UBI4/loxP-HIS1-loxP*::*ubi4*Δ*/ loxP-URA-loxP*::*ubi4*Δ, *NRG1-3xHA-ARG4*, *ADH1/adh1*::*Ptet-FLAG-230Q-RFP*	This study
CaLC4490 *ubi4*Δ/*ubi4*Δ +230Q-2	*ura3*Δ::*imm434/ura3*Δ::*imm434 arg4*Δ::*hisG/arg4*Δ::*hisG his1*Δ::*hisG/his1*Δ::*hisG*, *UBI4/loxP-HIS1-loxP*::*ubi4*Δ*/ loxP-URA-loxP*::*ubi4*Δ, *NRG1-3xHA-ARG4*, *ADH1/adh1*::*Ptet-FLAG-230Q-RFP*	This study

### Plasmid construction

To generate *C. albicans* strains expressing various lengths of Htt-PolyQ repeats, two approaches were taken based on the size of the repeats. FLAG-HTT-25PolyQ and FLAG-HTT-72PolyQ were PCR amplified from Met-FLAG-25PolyQ-CFP and Met-FLAG-72PolyQ-CFP (Duennwald *et al.* 2006b), using oligos oLC3001/3035. oLC3001 contained three mutations for CUG codon optimization of the Htt gene for *C. albicans*. In addition, RFP was PCR amplified from pLC435 (Keppler-Ross *et al.* 2008), using primers oLC3011/3036. A fusion PCR was then performed with oLC3001/3011 to fuse FLAG-HTT-25PolyQ or 72PolyQ with RFP. The product, along with the plasmid pNIM1, which contains the tetracycline ON promoter (Park and Morschhauser 2005), was digested with *Sal*I and *Bgl*II. Digested product and vector were ligated using T4 DNA ligase (NEB) and transformed into TOP10 cells (Invitrogen). Correct integration was tested for by PCR using oligos oLC3012/3013 (350 bp for 25PolyQ and 491 bp for 72PolyQ), and oLC3014/3015 (345 bp). This generated plasmids pLC774, tetON-FLAG-25PolyQ-RFP and pLC775, tetON-FLAG-72PolyQ-RFP. Plasmids were sequenced using oLC3012/3015.

Due to the nature of PolyQ repeats in generating 103Q and 230Q repeats, the FLAG-HTT-103PolyQ-RFP and FLAG-HTT-230PolyQ-RFP were synthesized (Invitrogen). The constructs and plasmid pNIM1 were liberated by digestion with *Bgl*II and *Sal*I, gel purified, ligated, and transformed into Stbl2 cells (Invitrogen). Upstream integration was tested by PCR using oligos oLC3012/3013, with an expected size of 583 bp for 103PolyQ and 964 bp for 230PolyQ. Downstream integration was tested by PCR using oligos oLC3014/3015 with an expected size of 345 bp. This generated plasmids pLC807, tetON-FLAG-103PolyQ-RFP and pLC814, tetON-FLAG-230PolyQ-RFP. Plasmids were sequence verified using oLC3012/3036/3014. Primers are listed in Supplemental Material, Table S1.

### Strain construction

*C. albicans* strains expressing 25Q and 72Q were generated by digesting pLC774 (25Q) or pLC775 (72Q) with *Apa*I and *Sac*II to liberate the cassette. Cassettes were transformed into CaLC3067. NAT-resistant transformants were PCR tested for correct integration at the *ADH1* locus by amplifying across both junctions using primer pairs oLC452/453 and oLC454/455. This generated CaLC3069 (25Q-1), CaLC3070 (25Q-2), CaLC3071 (72Q-1), and CaLC3072 (72Q-2).

To generate strains expressing 103Q or 230Q, pLC807 (103Q) or pLC814 (230Q) were digested with *Apa*I and *Sac*II to liberate the cassettes. Constructs were transformed into SN95 (CaLC239). NAT-resistant transformants were PCR tested for correct integration at the *ADH1* locus by amplifying across both junctions using primer pairs oLC452/453 and oLC454/455. This generated CaLC3252 (103Q-1), CaLC3253 (103Q-2), CaLC3256 (230Q-1), and CaLC3257 (230Q-2).

To generate an *hsp104*Δ/*hsp104*Δ mutant, the first allele of *HSP104* was deleted by PCR amplifying the NAT flipper cassette (pLC49) with oLC3967/3968 containing homology to sequence upstream and downstream of *HSP104*. The PCR product was transformed into SN95 (CaLC239) and NAT-resistant transformants were PCR tested with oLC275/3021 and oLC274/3969 to verify integration of the cassette. The NAT cassette was excised, and a second round of transformation was performed to delete the second allele, using the same PCR product as was used to delete the first allele. Verification of deletion was ascertained as described above, and loss of the wild-type allele was determined by PCR using oligos oLC782/785. The NAT cassette was then excised, generating CaLC4012. The 103Q and 230Q constructs were transformed into this background as described above, generating CaLC4088 (*hsp104*Δ/*hsp104*Δ + 103Q-1), CaLC4082 (*hsp104*Δ/*hsp104*Δ + 103Q-2), CaLC4083 (*hsp104*Δ/*hsp104*Δ + 230Q-1), and CaLC4084 (*hsp104*Δ/*hsp104*Δ + 230Q-2).

To generate an *sgt2*Δ/*sgt2*Δ mutant, the first allele of *SGT2* was deleted by PCR amplifying the NAT flipper cassette (pLC49) with oLC4197/4198 containing sequence homology upstream and downstream of *SGT2*. The PCR product was transformed into SN95 (CaLC239), and NAT-resistant transformants were PCR tested with oLC275/4199 and oLC274/4200 to verify integration of the cassette. The NAT cassette was excised, and a second round of transformation was performed to delete the second allele, using the same PCR product as was used to delete the first allele. Verification of deletion was ascertained as described above, and loss of the wild-type allele was determined by PCR using oligos oLC4201/4202. The NAT cassette was then excised, generating CaLC4385. The 103Q and 230Q constructs were transformed into this background as described above, generating CaLC4397 (*sgt2*Δ/*sgt2*Δ + 103Q-1), CaLC4398 (*sgt2*Δ/*sgt2*Δ + 103Q-2), CaLC4399 (*sgt2*Δ/*sgt2*Δ + 230Q-1), and CaLC4400 (*sgt2*Δ/*sgt2*Δ + 230Q-2).

To generate a *sis1*Δ/*sis1*Δ mutant, the first allele of *SIS1* was deleted by PCR amplifying the NAT flipper cassette (pLC49) with oLC4270/4271 containing sequence homology upstream and downstream of *SIS1*. The PCR product was transformed into SN95 (CaLC239) and NAT-resistant transformants were PCR tested with oLC275/4272 and oLC274/4273 to verify integration of the cassette. The NAT cassette was excised, and a second round of transformation was performed to delete the second allele, using the same PCR product as was used to delete the first allele. Verification of deletion was ascertained as previously described, and loss of the wild-type allele was determined using oligos oLC4378/4379. The NAT cassette was then excised, generating CaLC4450. The 103Q and 230Q constructs were transformed into this background as described above, generating CaLC4462 (*sis1*Δ/*sis1*Δ + 103Q-1), CaLC4463 (*sis1*Δ/*sis1*Δ + 103Q-2), CaLC4464 (*sis1*Δ/*sis1*Δ + 230Q-1) and CaLC4465 (*sis1*Δ/*sis1*Δ + 230Q-2).

A *ubi4*Δ/*ubi4*Δ mutant had already been generated in the BWP17 background (Leach *et al.* 2011). As such, the 103Q and 230Q constructs were transformed into this background as described above, generating CaLC4483 (BWP17 + 103Q-1), CaLC4484 (BWP17 + 103Q-2), CaLC4485 (BWP17 + 230Q-1) and CaLC4486 (BWP17 + 230Q-2), CaLC4487 (*ubi4*Δ/*ubi4*Δ + 103Q-1), CaLC4488 (*ubi4*Δ/*ubi4*Δ + 103Q-2), CaLC4489 (*ubi4*Δ/*ubi4*Δ + 230Q-1), and CaLC4490 (*ubi4*Δ/*ubi4*Δ + 230Q-2).

### Spotting and liquid assays

The spotting assay was performed with two independent strains of induced cells, grown at the temperature indicated, by determining OD_600_, diluting cells to an equal concentration, and spotting 10-fold dilutions (from ∼1 × 10^6^ cells/ml) onto YPD. Plates were imaged after 48 hr. All spotting assays were performed in duplicate on at least two separate occasions. The liquid assays were performed in flat bottom, 96-well microtiter plates (Sarstedt). Cell densities of overnight cultures were determined and dilutions were prepared such that ∼10^3^ cells were inoculated into each well. Assays were set up in a total volume of 0.2 ml/well of YPD or YPD containing 50 µg/ml doxycycline with twofold dilutions of the following drugs: Azetidine-2-carboxylic acid (Sigma, A0760), guanidine hydrochloride (Sigma, G4505), tunicamycin (Sigma, T7765), MG132 (Sigma, C2211), or geldanamycin (LC Labs, 4500). Plates were incubated in the dark at 30 or 42° for 48 hr, at which point plates were sealed and resuspended by agitation. Absorbance was determined at 600 nm using a spectrophotometer (Molecular Devices) and was corrected for background from the corresponding medium. Assays were performed in duplicate on at least two occasions. MIC data were quantitatively displayed with color using the program Java TreeView 1.1.3 (http://jtreeview.sourceforge.net).

### SDD-AGE

Strains were grown as described in the growth conditions section. Midlog phase cells were harvested, washed once with dH_2_O, and snap frozen in liquid nitrogen. Cells were lysed in 300 μl lysis buffer (100 mM Tris pH 7.5, 200 mM NaCl, 1 mM EDTA, 5% glycerol, 1 mM DTT, 8 mM PMSF, and protease inhibitor cocktail), and ∼200 mg of 0.5 mm acid-washed beads was added to each tube. Cells were mechanically disrupted on a Biospec Mini-Beadbeater for six 30 sec periods, with 1 min on ice between each cycle. A 26G syringe was used to puncture the bottom of the tube, and supernatant was collected by spinning for 1 min at 5000 rpm. Protein was quantified using a Bradford assay. Gel was prepared by dissolving 1.5% agarose in 1 × TAE and, once dissolved, SDS was added at 0.5%. Running buffer was 1 × TAE with 0.5% SDS. Samples were prepared in 4 × sample buffer (2 × TAE, 20% glycerol, 8% SDS, and bromophenol blue) at 1 × and boiled for 5 min at 100°. Samples were run at 50 V for ∼3.5 hr. Gels were rinsed in dH_2_O and then TBS (20 mM Tris pH 7.5 and 9 g NaCl up to a volume of 1 L in dH_2_O), and transferred onto PVDF membranes using Whatman: Turboblotter Rapid Downward Transfer Systems for 5 hr. Membranes were blocked and proteins detected as per the western blotting protocol.

### Microscopy

Imaging was performed on a Zeiss Imager M1 upright microscope and AxioCam MRm with AxioVision 4.7 software (Carl Zeiss, Inc.). An X-Cite series120 light source with ET HQ tetramethylrhodamine isothiocyanate (TRITC)/DsRED filter sets from ChromaTechnology (Bellows Falls, VT) was used for fluorescence microscopy.

### Dot blots

Dot blots were performed as described before (Cashikar *et al.* 2005). In brief, protein lysates were prepared using glass beads and lysis buffer [100 mM Tris PH 7.5, 200 mM NaCl, 1 mM EDTA, 5% glycerol, 1 mM DTT, 4 mM PMSF, and protease inhibitor tablet (Sigma)]. Lysates were normalized using BCA and diluted in phosphate-buffered saline (PBS) in threefold serial dilutions. All dilutions were loaded onto the BIO-DOT apparatus (Bio-Rad), and assembled with a 0.2 µm nitrocellulose membrane. A vacuum was applied to the manifold to transfer the lysate from the apparatus to the membrane. Membrane was then probed with a 1:5000 anti-FLAG antibody or 1:10,000 anti-PSTAIRE antibody and processed as a western blot.

### Quantitative RT-PCR

Cells were grown as stated in the growth conditions section. At midlog phase, strains were subjected to a 10 min 30–42° heat shock as described previously (Leach and Cowen 2014a), or left untreated at 30°. Cells were harvested from each culture, centrifuged at 3000 rpm for 2 min at 4°, and washed once with dH_2_O, before being snap frozen in liquid nitrogen and stored at −80°. RNA was subsequently isolated using the QIAGEN RNeasy kit and cDNA synthesis was performed using the AffinityScript cDNA synthesis kit (Stratagene). PCR was carried out using the SYBR Green JumpStart Taq ReadyMix (Sigma) with the following cycle conditions: 95° for 3 min, 95° for 10 sec and 60° for 30 sec for 39 rounds, 95° for 10 sec, and 65° for 5 sec. Reactions were performed in triplicate using the following primer pairs: *HSP104* (oLC1620/1621) and *HSP90* (oLC756/757). Transcript levels were normalized to *ACT1* (oLC2285/2286). Data were analyzed using the Bio-Rad CFX Manager software, version 3.1 (Bio-Rad).

### Western blotting

Total soluble protein was extracted and subjected to western blotting using published protocols (Leach and Cowen 2014a). Briefly, midlog-phase cells were pelleted by centrifugation, washed with sterile water, and resuspended in lysis buffer [0.1 M Tris-HCl (pH 8.0), 10% glycerol, 1 mM DTT, 1 mM PMSF, and protease inhibitor cocktail]. An equal volume of 0.5 mm acid-washed beads was added to each tube and cells were mechanically disrupted on a Biospec Mini-Beadbeater for six 30 sec periods, with 1 min on ice between each cycle. The lysate was pelleted by high-speed centrifugation and the supernatant removed for analysis. Protein concentration was determined by a Bradford assay. Protein samples were mixed with one-sixth volume of 6 × sample buffer [0.35 M Tris-HCl, 10% (wt/wt) SDS, 36% glycerol, 5% β-mercaptoethanol, and 0.012% bromophenol blue], and 75 μg protein was loaded in wells of an 8% SDS-PAGE gel. Separated proteins were transferred to a PVDF membrane for 1 hr at 100 V at 4°. Membranes were blocked in 5% milk in PBS containing 0.1% Tween 20 (PBS-T) at room temperature for 1 hr and subsequently incubated in a 1:10,000 dilution of anti-FLAG HRP-conjugated antibody (Sigma, A8592) in PBS-T + 5% milk [PBS 0.1% Tween-20 and 5% (w/v) milk]. Primary antibody was left on the membrane for 1 hr at room temperature. Membranes were washed in PBS-T and signals detected using an ECL western blotting kit as per the manufacturer’s instructions (Pierce).

### RNA-seq

Wild-type (CaLC239, SN95), 103Q (CaLC3252)-, and 230Q (CaLC3256)-expressing cells were grown in the absence or presence of 50 μg/ml doxycycline as described in the growth conditions section, and RNA was isolated using the Ambion RiboPure RNA purification yeast kit. Three biological replicates grown on three separate occasions were analyzed. RNA integrity was assayed on an Agilent Bioanalyser with freshly prepared gel-dye matrix according to the Agilent Bioanalyser RNA Pico protocol. Ribosomal RNA was removed using the Ribo-Zero Gold rRNA Removal Kit and TruSeq RNA-seq libraries were prepared according to the manufacturer’s instructions (Illumina), and sequenced on the Illumina HiSeq2000 platform. The sequencing data were assessed for quality using FastQC (http://www.bioinformatics.babraham.ac.uk/projects/fastqc/). All 51 bases for all 18 samples (six samples per biological replicate) had a minimum median Phred quality score over 32 (around 99.94% base call accuracy). The number of reads for each sample ranged from 12.3 M reads to 17.0 M reads with a minimum mapping rate of 95.9%. Each read was mapped against a *C. albicans* reference genome (SC5314, A21) using Tophat2 (version 2.1.0) with maximum mismatch of 1 and a gene transfer file (-N 1) (Kim *et al.* 2013), then expression of each RNA transcript was quantified and compared using cufflinks and cuffdiff (version 2.2.1) with the default setting (Trapnell *et al.* 2013). Both the reference genome and transcript annotation were downloaded from the *Candida* Genome Database (Inglis *et al.* 2012). All sequencing data are deposited at the Sequence Read Archive (Study Accession: SRP073330).

### Detection of polyQ interactors

Untagged wild-type (CaLC239, SN95) and 230Q (CaLC3256)-expressing cells were grown in the absence or presence of 50 μg/ml doxycycline, as described in the growth conditions section. Cells were harvested at an OD_600_ 0.8 at 4000 rpm for 10 min at 4°, washed twice with ice-cold 1 × PBS, and snap frozen in liquid nitrogen. Cells were resuspended in 10 ml of lysis buffer [20 mM Tris pH 7.5, 100 mM KCl, 5 mM MgCl, and 20% glycerol, with one protease inhibitor cocktail per 50 ml (complete, EDTA-free tablet, Roche Diagnostics), 1 mM PMSF (EMD Chemicals), and 0.5% Tween 20]. Cells were disrupted by bead-beating twice for 4 min with a 7 min break on ice between cycles. Lysates were centrifuged at 1300 × *g* for two 5 min cycles, recovering the supernatant at each stage. The combined lysate was centrifuged twice at 21,000 × g, first for 10 min and second for 5 min at 4°, and protein concentrations determined using the Bradford assay. Protein A/G beads (Santa Cruz SC-2003) were prepared for preclearing as per the manufacturer’s instructions, and protein samples were precleared for 1.5 hr at 4° with end-to-end rotation.

Anti-FLAG immunoprecipitations were performed by diluting protein samples to 20 mg/ml in lysis buffer containing 1 × Triton X-100, and incubating in 50 µl anti-FLAG M2 affinity gel (Sigma A2220) at 4° overnight with end-to-end rotation, as per the manufacturer’s specifications. Protein samples were eluted by washing beads twice with lysis buffer with detergent followed by three times with lysis buffer without detergent. Fresh elution buffer (9.5 M NH_4_OH, pH 11.0–12.0 and 0.5 mM EDTA) was then added at three times the bead volume and samples incubated at 4° for 15 min with end-over-end rotation. This was repeated three times. Samples were digested in-solution as described previously (Gundry *et al.* 2009). Briefly, DTT was added to a final concentration of 10 mM and samples were incubated with end-over-end rotation at room temperature for 30 min. Trypsin (Promega, MS grade) was added and samples were incubated overnight at 37° with end-over-end rotation. Reactions were stopped by adding 5 μl of 1.0% TFA.

Samples were analyzed on an Orbitrap analyzer (Q-Exactive, Thermo Fisher Scientific, San Jose, CA) outfitted with a nanospray source and EASY-nLC nano-LC system (Thermo Fisher Scientific). Lyophilized peptide mixtures were dissolved in 11 μl of 0.1% formic acid and 5 μl were loaded onto a 75 μm × 50 cm PepMax RSLC EASY-Spray column filled with 2 μM C18 beads (Thermo Fisher Scientific) at a pressure of 800 Bar. Peptides were eluted over 60 min at a rate of 250 nl/min using a 0–35% acetonitrile gradient in 0.1% formic acid. Peptides were introduced by nano-electrospray into the Q-Exactive mass spectrometer (Thermo Fisher Scientific). The instrument method consisted of one MS full scan (400–1500 m/z) in the Orbitrap mass analyzer with an automatic gain control (AGC) target of 1e6, maximum ion injection time of 120 msec, and a resolution of 70,000 followed by 10 data-dependent MS/MS scans with a resolution of 17,500, an AGC target of 1e6, maximum ion time of 120 msec, and one microscan. The intensity threshold to trigger a MS/MS scan was set to 1.7e4. Fragmentation occurred in the HCD trap with normalized collision energy set to 27. The dynamic exclusion was applied using a setting of 10 sec.

### Database searching

Tandem mass spectra were extracted, charge state deconvoluted, and deisotoped by Xcalibur version 2.2. All MS/MS samples were analyzed using Sequest (Thermo Fisher Scientific; version 1.4.1.14) and X! Tandem [The GPM, thegpm.org; version CYCLONE (2010.12.01.1)]. Sequest was set up to search Uniprot-C_albicans.fasta (downloaded Feb 28 2014, 9082 entries) assuming the digestion enzyme trypsin. X! Tandem was set up to search the Uniprot-C_albicans database (downloaded Feb 28 2014, 9082 entries) also assuming trypsin. Sequest and X! Tandem were searched with a fragment ion mass tolerance of 0.020 kDa and a parent ion tolerance of 10.0 PPM. Deamidated of asparagine and glutamine, oxidation of methionine, and carbamidomethyl of cysteine were specified in Sequest as variable modifications. Glu->pyro-Glu of the N-terminus, ammonia-loss of the N-terminus, Gln->pyro-Glu of the N-terminus, deamidated of asparagine and glutamine, oxidation of methionine, and carbamidomethyl of cysteine were specified in X! Tandem as variable modifications.

### Protein identification

Scaffold (version Scaffold_4.5.0, Proteome Software Inc., Portland, OR) was used to validate MS/MS-based peptide and protein identifications. Peptide identifications were accepted if they could be established at > 95.0% probability. Peptide Probabilities from Sequest were assigned by the Scaffold Local FDR algorithm. Peptide Probabilities from X! Tandem were assigned by the Peptide Prophet algorithm (Keller *et al.* 2002) with Scaffold Δ-mass correction. Protein identifications were accepted if they could be established at > 95.0% probability, contained at least one identified peptide, and were present in both biological replicates. Protein probabilities were assigned by the Protein Prophet algorithm (Nesvizhskii *et al.* 2003). Proteins that contained similar peptides and could not be differentiated based on MS/MS analysis alone were grouped to satisfy the principles of parsimony. Proteins sharing significant peptide evidence were grouped into clusters. The mass spectrometry data has been deposited into the Proteome Exchange PRIDE database (http://www.proteomexchange.org/) under the accession number PXD003916.

### Data availability

All sequencing data are deposited at the Sequence Read Archive (Study Accession: SRP073330). The mass spectrometry data has been deposited into the Proteome Exchange PRIDE database (http://www.proteomexchange.org/) under the accession number PXD003916. 

## Results

### Absence of polyQ aggregation and toxicity in C. albicans

We generated a series of constructs containing human Htt exon 1 with varying lengths (25Q, 72Q, 103Q, and 230Q) of polyQ tracts (CAG repeats) with an amino-terminal FLAG tag and a C-terminal Red Fluorescent Protein (RFP), under the control of the tetracycline ON promoter, integrated at the *ADH1* locus in *C. albicans* (Figure S1A) (Park and Morschhauser 2005). Our initial studies focused on 25Q and 72Q, where we examined cells for aggregation and toxicity; 25Q represents a nondisease related, nontoxic, and nonaggregating polyQ length, and 72Q represents a highly aggregation-prone and toxic polyQ length in many different model organisms including *S. cerevisiae* (Figure S1B), *D. melanogaster*, *C. elegans*, cultured mammalian cells, and rodents (Burright *et al.* 1995; Morley *et al.* 2002; Waelter *et al.* 2001; Marsh *et al.* 2000).

First, we examined PolyQ aggregation by microscopy in cells that were grown to midlog phase in the presence of 50 μg/ml doxycycline to induce polyQ expression (Figure S1A). Addition of doxycycline caused cells containing the polyQ expansions to fluoresce, with the signal uniformly and evenly distributed throughout the cell for both 25Q and 72Q. The lack of polyQ aggregation for 72Q suggests that *C. albicans*, in contrast to *S. cerevisiae* and many other organisms, is resistant to polyQ aggregation.

Further, we examined polyQ toxicity in *C. albicans*. Wild-type, 25Q-, and 72Q- expressing cells were grown at 30 or 42° in the absence and presence of doxycycline to induce expression of the expanded polyQ regions, and spotted on YPD plates to assess toxicity (Figure S1C). No growth defect was observed for any of the strains. Next, we monitored growth in 96-well plates in the absence and presence of doxycycline with a gradient of the nucleoside antibiotic tunicamycin, which induces ER stress and the UPR, and increases polyQ toxicity in *S. cerevisiae* and mammalian cells (Wimalasena *et al.* 2008). Comparable growth was observed for two independent 25Q- and 72Q-expressing strains and the wild-type strain under all conditions (Figure S1D). Thus, expression of 72Q does not elicit polyQ toxicity even in the context of ER stress. This is in contrast to *S. cerevisiae*, for which induction of 72Q using galactose causes toxicity compared to wild-type or 25Q-expressing strains (Figure S1E) (Duennwald *et al.* 2006b).

**Figure 1:**
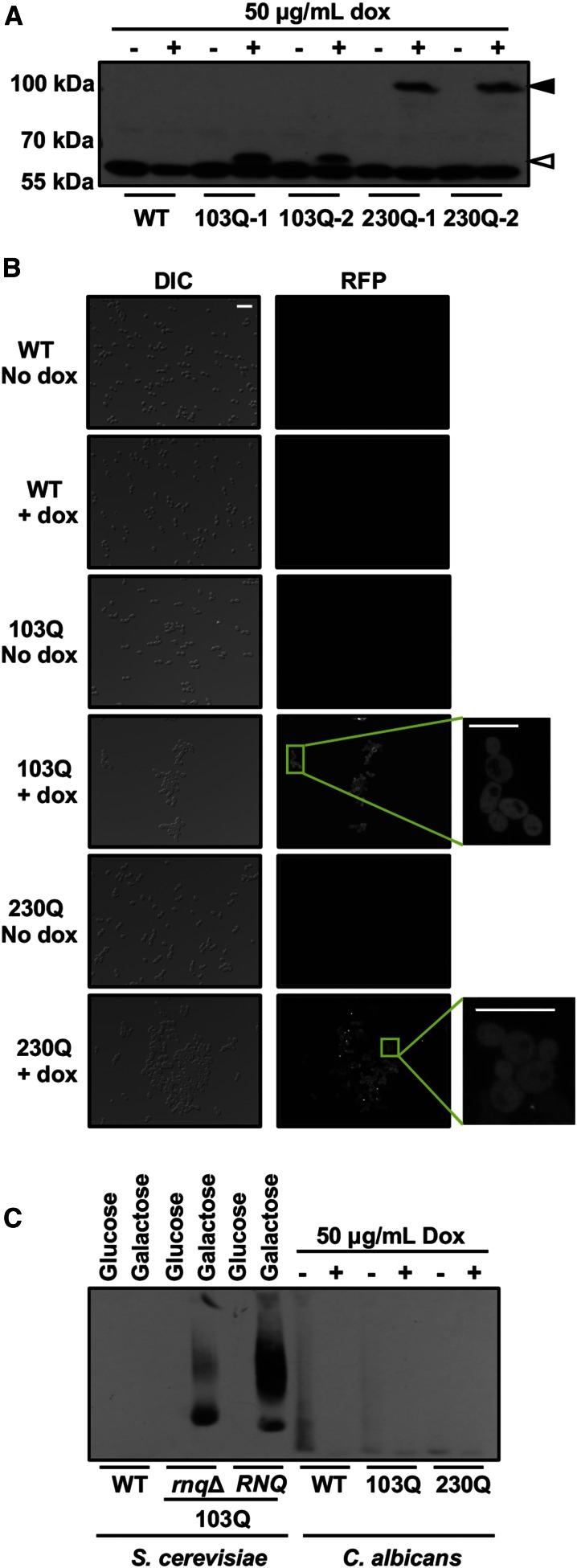
Expression of up to 230Q does not cause aggregation in *C. albicans*. (A) Western blot analysis using the FLAG epitope of wild-type (WT) and two independent strains harboring 103Q or 230Q expression constructs under the control of the tetracycline ON promoter. Protein expression was monitored after a total of 30 hr induction [−, no doxycycline (dox); +, 50 μg/ml dox]. White arrow = 103Q, black arrow = 230Q. (B) Live cell red fluorescent protein (RFP) (middle) and differential interference contrast (DIC) (left) microscopy of WT, and strains harboring 103Q or 230Q expression constructs in the absence (No dox) or presence (+ dox) of 50 μg/ml dox. Inset: magnification of a subset of cells representing polyQ expression. Scale bar, 20 μm. (C) Semidenaturating detergent agarose gel electrophoresis analysis of aggregated proteins expressed from *S. cerevisiae* WT, 103Q in *rnq*Δ, and 130Q in [*RNQ*^+^] grown in minimal medium with glucose (not inducing) or galactose (inducing) (lanes 1–6), *C. albicans* WT, and strains harboring 103Q or 230Q expression constructs grown in yeast peptone dextrose medium in the absence (−, not inducing) or presence (+, inducing) of 50 μg/ml dox. Detection via the N-terminal FLAG-tag.

Next, we examined very long polyQ expansions, up to 230Q. We assessed polyQ expression by western blot in the absence (no induction) and presence (induction) of doxycycline. In two independent strains for 103Q and 230Q, we observed a clear signal in the presence of doxycycline (Figure 1A), confirming that the polyQ repeats are expressed. This also indicates solubility of the polyQ expansion proteins in *C. albicans*, in contrast to polyQ expression in *S. cerevisiae* and other organisms, for which polyQ expansions are unable to migrate in SDS-PAGE due to insolubility (Krobitsch and Lindquist 2000). To further study the aggregation of these very long polyQ expansions, we performed fluorescence microscopy to visualize RFP (Figure 1B). The fluorescence signal for the 103Q constructs was once again uniformly distributed within cells, with no obvious punctate structures visible (Figure 1B). Only 8% of cells expressing 230Q displayed some punctate structures, possibly indicating rare aggregation beginning in the few cells over the 30 hr time course of protein induction (Figure 1B).

In order to corroborate our microscopy results, we performed SDD-AGE assays to characterize polyQ aggregation biochemically in the 103Q- and 230Q-expressing strains. We included the *rnq*Δ and [*RNQ*^+^] 103Q polyglutamine-expanded Htt strains from *S. cerevisiae*, in which strains harboring as short a polyQ expansion as 46Q are known to be insoluble (Alberti *et al.* 2009; Chafekar *et al.* 2012). Rnq1 in its prion confirmation plays an essential role in polyQ aggregation, leading to toxicity (Meriin *et al.* 2002). Since *C. albicans* lacks prion proteins, we included the *rnq*Δ as a suitable control. In stark contrast to the insoluble protein aggregates observed in *S. cerevisiae* 103Q-expressing strains, the 103Q and even 230Q expansions remained completely soluble in *C. albicans* (Figure 1C). Thus, in *C. albicans*, polyQ expansions do not form aggregates detectable by microscopy or biochemical assays, even with extremely long polyQ stretches that would readily aggregate in many other eukaryotic cell types.

**Figure 2:**
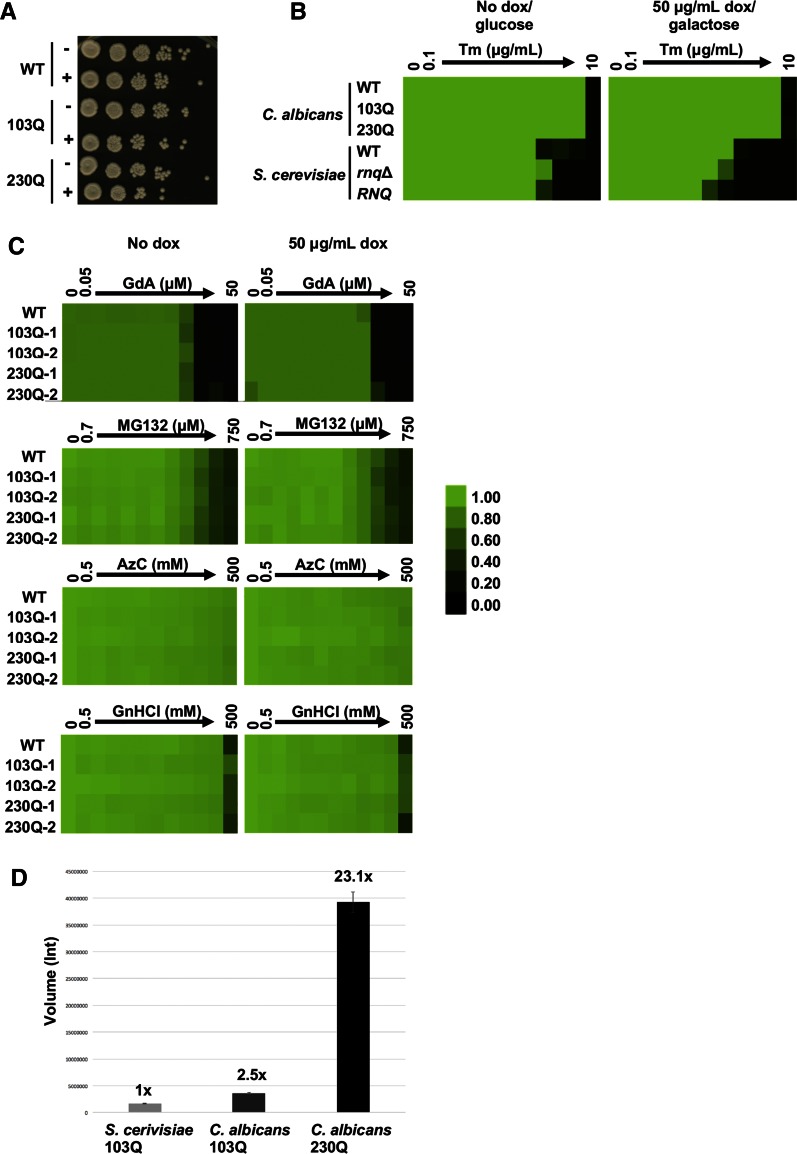
Expression of up to 230Q in combination with proteotoxic stressors does not cause toxicity in *C. albicans*. (A) Wild-type (WT) and strains harboring 103Q or 230Q expression were monitored after a total of 30 hr induction (−, no doxycycline (dox); +, 50 μg/ml dox) before being serially diluted ten-fold and spotted onto YPD solid medium, grown at 30°, and imaged after 48 hr. (B) Toxicity of *S. cerevisiae* WT, 103Q in *rnq*Δ, and 103Q in [*RNQ*^+^] compared to *C. albicans* WT and strains harboring 103Q or 230Q expression constructs was assessed in the presence of a tunicamycin (Tm) gradient from 0 to 10 μg/ml, in twofold dilutions in YPD with (inducing) or without (not inducing) 50 μg/ml dox or minimal medium with glucose (not inducing) or galactose (inducing). Growth was measured after 48 hr static incubation by absorbance at 600 nm and normalized relative to the no Tm control. For each strain, optical densities were averaged for duplicate measurements and displayed quantitatively using Treeview, as shown in the color bar. Data are representative for three biological replicates. (C) Toxicity of the *C. albicans* strains harboring 103Q and 230Q was assessed in the presence of proteotoxic stressors. Gradients were performed in twofold dilutions, and strains set up in YPD in the absence (no dox) or presence of 50 μg/ml dox. Growth was measured after 48 hr static incubation at 30° by absorbance at 600 nm and normalized relative to the untreated control. AzC, azetidine-2-carboxylic acid; GdA, geldanamycin; GnHCl, guanidine hydrochloride. (D) Dot blots were performed to determine the expression levels of 103Q in *S. cerevisiae*, and 103Q and 230Q in *C. albicans*. Protein lysates were serially diluted in PBS and loaded on the dot blot apparatus assembled with a nitrocellulose membrane. Blot quantification was carried out using Bio-Rad Image Lab 5.2 software following the blot scan. PolyQ expression levels have been normalized to the corresponding loading control (PSTAIRE). *S*. *cerevisiae* 103Q was set to 1. 103Q expression in *C. albicans* is 2.5 times greater than in *S. cerevisiae*, and 230Q is 23 times greater. AzC, azetidine-2-carboxylic acid; GdA, geldanamycin; GnHCl, guanidine hydrochloride.

We next tested whether the expression of 103Q or 230Q causes polyQ toxicity, as evidenced by reduced growth in *C. albicans*. PolyQ expansions were induced by growing cells for 24 hr, followed by subculture and growth for an additional 6 hr before plating cells on YPD in serial dilutions to assess potential growth defects (Figure 2A). All strains grew at similar rates, suggesting that expression of polyQ proteins with long glutamine regions is not toxic to *C. albicans* (Figure 2A).

In an attempt to unmask polyQ toxicity, we expressed the polyQ expansion proteins in the presence of additional proteotoxic stresses. First, we set up a growth assay in 96-well plates in the presence of doxycycline to express the expanded polyQ regions with a concentration gradient of tunicamycin. We included strains in the absence of doxycycline and the *S. cerevisiae* wild-type, *rnq*Δ, and [*RNQ*^+^] 103Q polyglutamine-expanded Htt strains as controls. As expected, expression of the 103Q polyglutamine-expanded Htt in *S. cerevisiae* [*RNQ*^+^] strain impaired growth compared to wild-type cells lacking the polyQ expansion, with a minor effect seen in the *rnq*Δ strain (Figure 2B). In stark contrast, neither the *C. albicans* 103Q or 230Q strains exhibited any growth defect (Figure 2B). We further tested other compounds that perturb protein homeostasis, including the Hsp90 inhibitor geldanamycin, the proteasome inhibitor MG132, the protein unfolding inducer azetidine-2-carboxylic acid, and the Hsp104 inhibitor guanidine hydrochloride (Figure 2C). Even at very high concentrations, all except geldanamycin had no impact on the growth of wild-type cells. Expression of up to 230Q had no additional effect on cell growth, demonstrating that *C. albicans* does not display polyQ toxicity even in the presence of proteotoxic stress (Figure 2C). To ensure that polyQ Htt protein levels were similar between *S. cerevisiae* and *C. albicans*, we compared polyQ Htt proteins using dot blots. The 103Q polyglutamine-expanded protein levels in *S. cerevisiae* were comparable to the expression levels of 103Q in *C. albicans*. However, 230Q expression levels in *C. albicans* were more than 20-fold higher than 103Q in *S. cerevisiae*, ruling out the possibility that the lack of toxicity in *C. albicans* could be due to low levels of polyQ expression (Figure 2D).

### High temperature induces polyQ aggregation independent of toxicity

When cells are subjected to a heat shock or continuous growth at high temperatures, many proteins unfold, which reduces growth and general fitness (Leach *et al.* 2012a). We asked whether polyQ expression combined with growth at high temperature would overwhelm the protein folding machinery, leading to increased aggregation and toxicity. First, we examined aggregation of our polyQ expansions by fluorescence microscopy. PolyQ expansions were induced by growing cells for 24 hr, followed by subculture and growth for an additional 6 hr at 42° before imaging. The fluorescence signal for the 103Q constructs was once again evenly distributed throughout the cell, with only minimal punctate structures visible (Figure S2A). In contrast, induction of 230Q produced extensive punctate structures, indicating that the longer polyQ expansions no longer remain soluble and aggregate.

Based on this result and previously published work, we postulated that expressing 103Q and 230Q at high temperature with a gradient of the unfolded protein stressor tunicamycin would unmask polyQ toxicity. To our surprise, once again, none of the polyQ expressing strains exhibited any growth defect (Figure S2B). We also tested the unfolded protein inducer azetidine-2-carboxylic acid and the Hsp104 inhibitor guanidine hydrochloride on cells incubated at 42°. Again, we observed no toxicity whatsoever in any of the polyQ expressing strains (Figure S2C).

### Expression of polyglutamine-expanded Htt has no effect on the global transcriptional response

**Figure 3:**
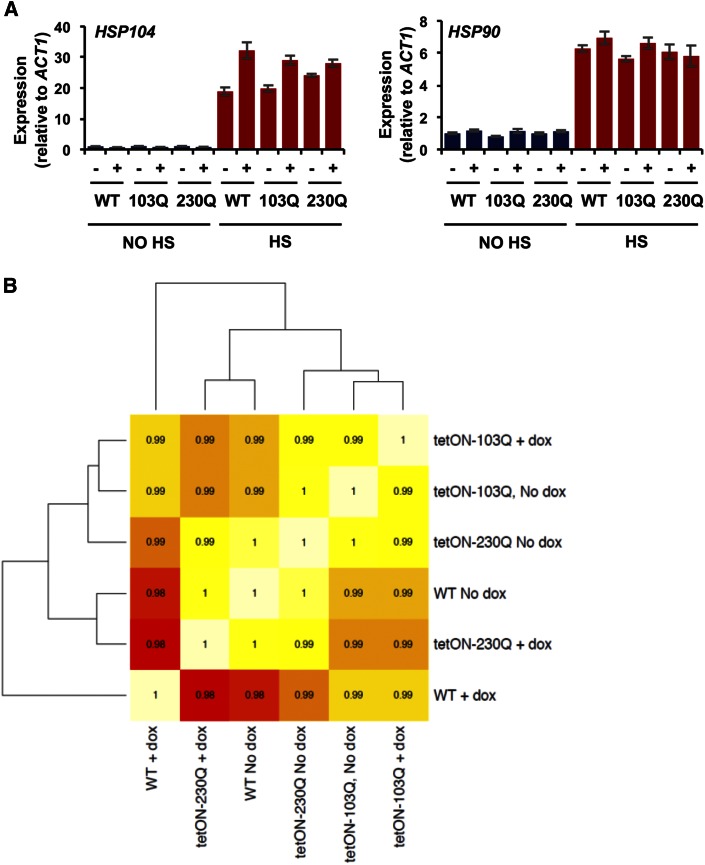
Expression of polyQ does not affect the transcriptome of *C. albicans*. (A) *C. albicans* wild-type (WT) and strains harboring 103Q or 230Q expression constructs were grown at 30° (no heat shock [HS]) or subjected to a 10 min 30–42° HS, in the absence (−) and presence (+) of 50 μg/ml doxycycline (dox) to induce polyQ expression; transcript levels of target genes were measured and normalized to the *ACT1* loading control. Data represent mean values ± SD from two independent biological replicates. (B) Heat map illustrating the Pearson correlation of RNA sequencing (RNA-seq) global gene expression between WT and strains harboring 103Q or 230Q expression constructs in the absence and presence of 50 μg/ml dox to induce polyQ expression.

Studies in cultured mammalian cells, yeast, and mice, have documented a strong transcriptional response to the expression of polyQ expansion proteins (Hughes *et al.* 2001; Mohan *et al.* 2014). Given our findings of the exceptional resistance of *C. albicans* to polyQ aggregation and toxicity, we asked whether the cells effectively upregulate protective genes, such as molecular chaperones, to prevent polyQ aggregation and toxic consequences. Specifically, we tested whether polyQ expansion proteins activate the HSR, leading to upregulation of key molecular chaperones that could modulate polyQ proteins. To this end, we expressed 103Q and 230Q expansions by growing cells for 24 hr, followed by subculture and growth for an additional 6 hr before subjecting cells to a short 10 min 42° heat shock. Expression of *HSP90*, a key molecular chaperone known to aid in the folding of ∼10% of the proteome (Leach *et al.* 2012b), and *HSP104*, which functions as a potent protein disaggregase (Grimminger-Marquardt and Lashuel 2010), was then examined. In the absence of heat shock, there was no difference in *HSP90* or *HSP104* transcript levels between wild-type and polyQ-expressing strains (Figure 3A). Heat shock induced transcript levels of both genes, as expected, but once again no significant difference was observed between wild-type and polyQ expansion strains (Figure 3A). These experiments indicate that polyQ expression does not induce a HSR under normal growth conditions, and that *C. albicans* cells expressing polyQ proteins elicit a normal HSR, in contrast to *S. cerevisiae* and mammalian cells.

In addition, we probed global transcriptional changes using RNA-seq to determine the response to 103Q and 230Q expression. PolyQ expression was induced for a total of 30 hr before RNA extraction and sequencing. Surprisingly, very few gene expression differences were observed between wild-type, 103Q and 230Q data sets (Figure 3B). Indeed, in each of three replicates performed, the correlation coefficient between any two samples remained above 0.88 (Figure S3), with few genes being up or downregulated in any of the conditions tested (File S1). The data document that expression of polyQ proteins has little or no effect on gene expression in *C. albicans*.

### Sis1 and Sgt2 interact with polyglutamine-expanded Htt

**Figure 4:**
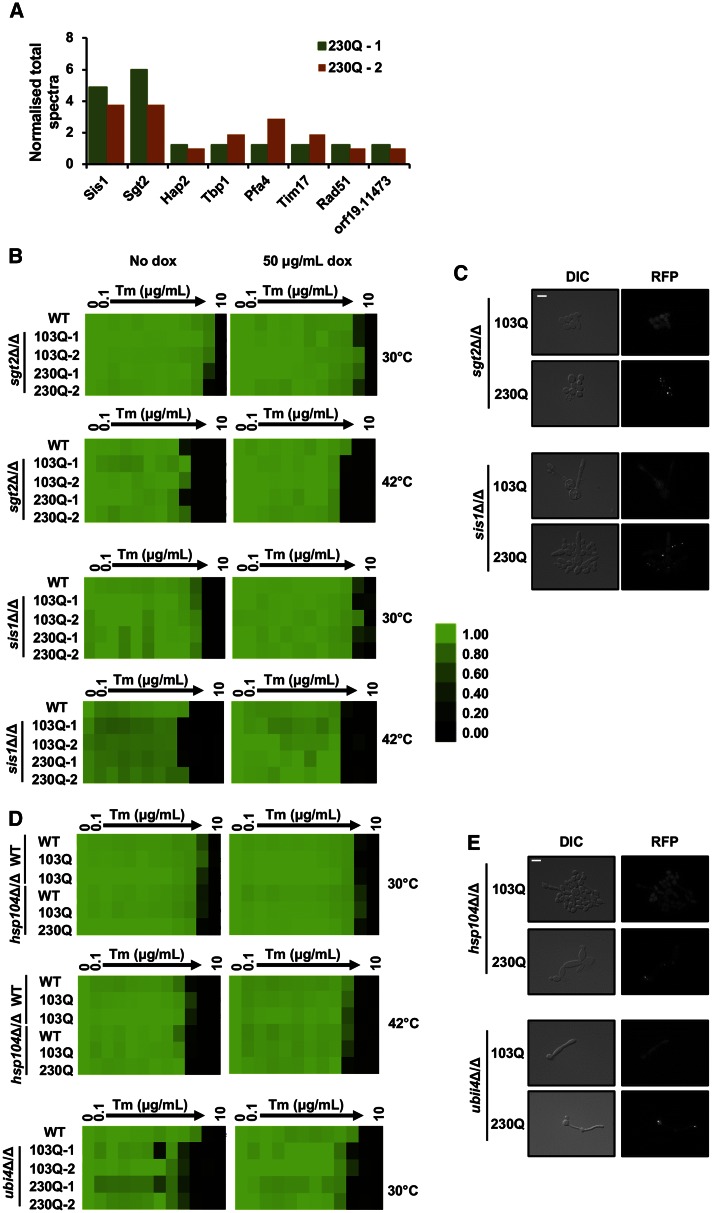
Deletion of polyQ protein interactors does not increase toxicity or aggregation. (A) Protein interactors of 230Q. Histogram depicting the normalized total spectra count of proteins found to interact with 230Q in two biological replicates (230Q-1 and 230Q-2). (B) Toxicity in wild-type (WT) and independent *sis1*Δ/*sis1*Δ or *sgt2*Δ/*sgt2*Δ mutants, harboring 103Q or 230Q expression constructs, was assessed in the presence of a tunicamycin (Tm) gradient from 0 to 10 μg/ml, in twofold dilutions in YPD with or without 50 μg/ml doxycycline (dox) at 30 or 42°. Growth was measured after 48 hr of static incubation by absorbance at 600 nm and normalized relative to the no Tm control. For each strain, optical densities were averaged for duplicate measurements and displayed quantitatively using Treeview, as shown in the color bar. Data are representative for three biological replicates. (C) Live cell microscopy of 103Q and 230Q in *sis1*Δ/*sis1*Δ or *sgt2*Δ/*sgt2*Δ mutants grown at 42°. Scale bar, 10 μm. (D) Toxicity of WT and independent *hsp104*Δ/*hsp104*Δ or *ubi4*Δ/*ubi4*Δ mutants harboring 103Q or 230Q expression constructs was assessed in the presence of a Tm gradient from 0 to 10 μg/ml, in twofold dilutions in YPD with or without 50 μg/ml dox at 30 or 42°. Growth was measured after 48 hr of static incubation by absorbance at 600 nm and normalized relative to the no Tm control. For each strain, optical densities were averaged for duplicate measurements and displayed quantitatively using Treeview, as shown in the color bar. Data are representative for three biological replicates. (E) Live cell microscopy of 103Q and 230Q in *hsp104*Δ/*hsp104*Δ or *ubi4*Δ/*ubi4*Δ mutants grown at 42°. Scale bar, 10 μm. DIC, differential interference contrast; RFP, red fluorescent protein.

The negligible transcriptional response to polyQ expansions suggested that *C. albicans* cells cope with high levels of polyQ proteins with minimal changes in gene expression programs. Therefore, we explored a mass spectrometry-based approach to determine which proteins interact with the polyQ proteins using 230Q as an example. IP-mass spectrometry showed that only eight proteins are enriched in the 230Q strain upon induction of the polyQ expansion, compared to the uninduced condition and the wild-type strain (Table 2). Interestingly, the molecular chaperones Sgt2 and Sis1 had among the strongest interactions with the 230Q expansion (Figure 4A). Sgt2 has previously been implicated as an amyloid sensor, regulating the targeting of chaperones to aggregation-prone proteins in *S. cerevisiae* (Kiktev *et al.* 2012). Furthermore, Sis1 has recently been found to be sequestered by polyQ aggregates, which in turn inhibits degradation of misfolded proteins via the ubiquitin–proteasome system (Park *et al.* 2013). To determine if these proteins prevent polyQ aggregation and toxicity, we generated deletion mutants for each of them in *C. albicans*, and expressed our 103Q and 230Q expansions. We then tested polyQ toxicity using a growth assay with an increasing concentration of tunicamycin at either 30 or 42° (Figure 4B). In both *sis1* and *sgt2* deletion mutants, no polyQ toxicity was observed compared to wild-type strains (Figure 4B). We also explored polyQ aggregation via fluorescence microscopy after growth at 42° (Figure 4C). We found that the 103Q expansion remains evenly distributed throughout the cell, indicative of a soluble polyQ protein. Yet when the 230Q expansion is expressed at higher temperatures, we observe a few punctate structures forming, indicative of aggregation, similar to our experiment in heat-shocked wild-type cells (Figure S2A).

**Table 2:** PolyQ interactors

Protein	Role	PolyQ	PolyN
Sis1	Putative type II HSP40 cochaperone	N/A	N/A
Sgt2	Putative small tetratricopeptide repeat (TPR)-containing protein	N/A	N/A
Hap2	CCAAT-binding transcription factor; regulates low-iron induction of FRP1	13	9
Tbp1	Transcription initiation factor; binds TATA box sequence	N/A	N/A
Pfa4	Palmitoyltransferase with autoacylation activity; required for palmitoylation of amino acid permeases	N/A	N/A
Tim17	Predicted component of the Translocase of the Inner Mitochondrial membrane (TIM23 complex), involved in protein import into mitochondria	N/A	N/A
Rad51	Protein involved in homologous recombination and DNA repair	N/A	N/A
Orf19.11473	Ortholog(s) have DNA-directed RNA polymerase activity, RNA polymerase III activity and role in tRNA (transfer RNA) transcription from RNA polymerase III promoter, transcription initiation from RNA polymerase III promoter	N/A	N/A

N/A, not applicable.

Next, we tested the impact of additional key regulators of protein homeostasis on polyQ toxicity and aggregation. Given the established role of Hsp104 in modulating polyQ aggregation and toxicity, we assessed the impact of *HSP104* deletion in *C. albicans* on the aggregation and toxicity of 103Q and 230Q. In addition, we postulated that the ubiquitin-proteasome pathway may also play a role by targeting the expansions for efficient degradation, preventing any cellular toxicity. Thus, we also used a deletion of *UBI4*, the gene encoding polyubiquitin. Once again, we subjected the strains to ER stress via tunicamycin treatment and performed growth assays at 30 or 42°. Notably, the *ubi4*Δ/*ubi4*Δ is temperature-sensitive, and we could only test this mutant at 30° (Leach *et al.* 2011). As with *SIS1* and *SGT2*, deletion of *HSP104* or *UBI4* did not exacerbate growth defects upon expression of 103Q and 230Q (Figure 4D). Again, we performed fluorescence microscopy to determine if any further aggregation was occurring in the mutants. Cells were grown at 42° for a short period of time to ensure that viability of the temperature-sensitive mutants was not affected, and then cells were imaged. Similar to our other mutants, 103Q remained evenly distributed across the cytosol and 230Q formed few punctate structures (Figure 4E).

**Figure 5:**
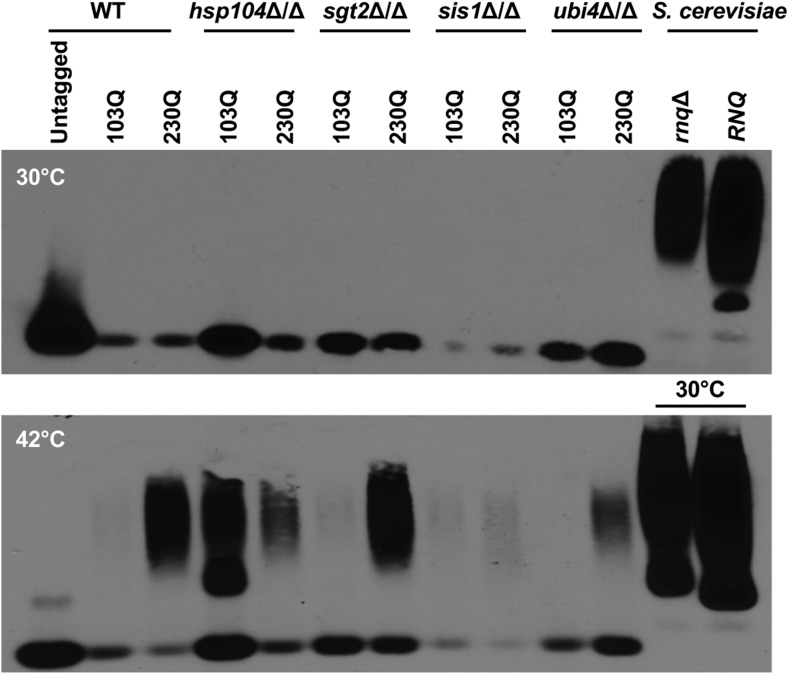
Expression of up to 230Q in mutants involved in protein quality control does not cause aggregation in *C. albicans*. Semidenaturating detergent agarose gel electrophoresis analysis of aggregated proteins expressed from *C. albicans* wild type (WT) and different mutant backgrounds harboring 103Q or 230Q expression constructs at 30° (top) *vs.* 42° (bottom). *S. cerevisiae* 103Q in *rnq*Δ and [*RNQ*^+^] were grown at 30° only and were included as a control. Detection via the N-terminal FLAG-tag.

Finally, we corroborated these data by performing SDD-AGE assays in each of the deletion strains at 30 and 42°. PolyQ expansions were induced in strains grown for 24 hr, followed by subculturing for an additional 6 hr at 30 or 42°. At 30°, polyQ aggregates were observed in the *S. cerevisiae* 103Q expressing *rnq*Δ and [*RNQ*^+^] strains, but not in any of the *C. albicans* strains (Figure 5). However, there was considerable aggregation observed in most of our *C. albicans* mutants expressing 230Q grown at 42°, as well as in the *hsp104*Δ*/hsp104*Δ mutant expressing 103Q. Due to the temperature sensitivity of *S. cerevisiae*, aggregation of our *C. albicans* mutants at 42° was compared to our *S. cerevisiae* strains grown at 30°, whereby we still observed significantly lower levels of aggregation (Figure 5).

## Discussion

PolyQ expansion proteins, their misfolding, aggregation, and toxicity are closely associated with at least nine neurodegenerative diseases (Fan *et al.* 2014). In addition, polyQ expansion proteins have successfully served as experimental paradigms to explore basic mechanisms by which cellular defense systems modulate the toxicity often associated with protein misfolding. Following this logic, we established a *C. albicans* model expressing polyQ expansion proteins to gain insights into the protein quality control system and stress response programs of this major human fungal pathogen. However, we found that the expression of amino-terminal fragments of a polyQ-expanded huntingtin protein (103Q and 230Q) in *C. albicans* does not share any of the previously established biochemical and cellular hallmarks of polyQ expansion proteins and their interactions with cellular protein control systems.

The amino acid sequence of a protein determines its propensity to misfold and aggregate, with sequences rich in asparagine and glutamine favoring aggregation (Alberti *et al.* 2009). Indeed, in *S. cerevisiae*, cultured mammalian cells, *D. melanogaster*, *C. elegans*, rodents, and many other models, expression of polyQ expansion proteins leads to considerable aggregation in the cytosol or the nucleus (Brignull *et al.* 2006; Bradford *et al.* 2010; Faber *et al.* 1999; Jackson *et al.* 1998; Duennwald *et al.* 2006b; Duennwald 2011), which is often accompanied by reduced cellular and organismal fitness due to impaired core cellular functions, particularly those involved in protein quality control. Strikingly, our findings clearly demonstrate that *C. albicans* cells expressing polyQ expansion proteins had no polyQ aggregation under basal physiological conditions, and there was negligible polyQ toxicity or transcriptional response, with the HSR also being unaffected. This result is striking given that the *C. albicans* polyQ constructs are expressed at higher levels than the corresponding polyQ constructs in *S. cerevisiae* (Figure 2D).

**Figure 6:**
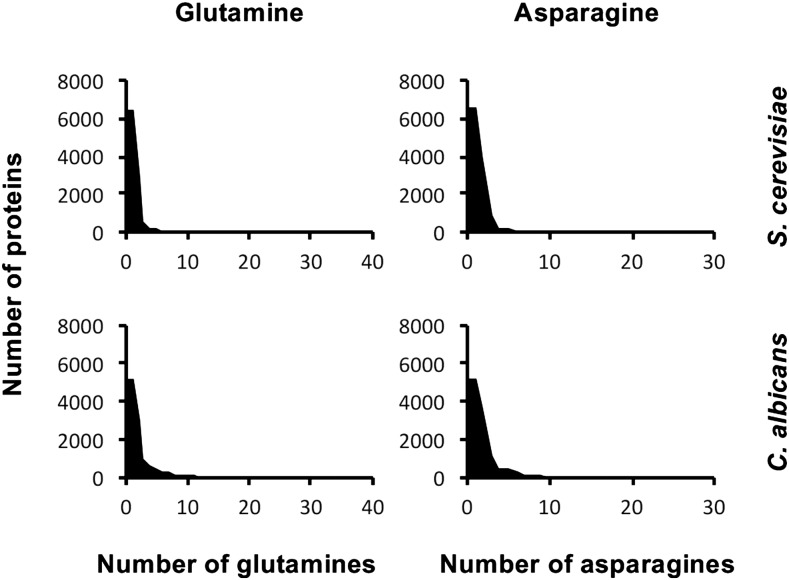
Bioinformatic analysis reveals similar features between *S. cerevisiae* and *C. albicans* Q/N-rich proteome. Length distribution of glutamine and asparagine runs in *S. cerevisiae* and *C. albicans*, with both species containing one protein with ≤40 glutamines in a row or ≤30 asparagines in a row.

This lack of toxicity associated with polyQ expansions resonates with recent findings in *D. discoideum*, an organism with a proteome that is highly enriched in polyN and polyQ proteins (Malinovska *et al.* 2015; Santarriaga *et al.* 2015). In *D. discoideum*, polyQ aggregation could only be induced by exposure to strong proteotoxic stress conditions, such as a heat shock. This was interpreted as evidence that organisms with proteomes enriched in aggregation-prone proteins, such as polyQ expansion proteins, have evolved highly effective protein quality control systems to prevent the toxic consequences of protein misfolding. Notably, our results in *C. albicans*, along with studies performed in other organisms, such as *S. pombe* and *D. melanogaster*, do not fall in line with this correlation. Unlike *D. discoideum*, the proteome of *C. albicans* does not have a high proportion of aggregation-prone proteins (polyQ expansion and other proteins) (Figure 6), and *S. pombe* contains very few aggregation-prone proteins in its proteome (Schaefer *et al.* 2012). Yet both yeasts show little to no polyQ aggregation and polyQ toxicity. In fact, even when we challenge *C. albicans* protein quality control systems by genetic manipulation or exposure to proteotoxic stress conditions, *C. albicans* exhibits little polyQ aggregation and no polyQ toxicity. Further, the proteome of *D. melanogaster*, similar to the proteome of *D. discoideum*, is highly enriched in Q- and N-rich aggregation-prone proteins (Schaefer *et al.* 2012). Yet unlike *D. discoideum*, the expression of polyQ expansion proteins results in polyQ aggregation and toxicity in fly cells (Jackson *et al.* 1998). PolyQ-expanded proteins engage in many unusually stable protein–protein interactions and often sequester other proteins rich in N or Q repeats, which can impair their functions, resulting in cellular toxicity (Duennwald *et al.* 2006a; Kochneva-Pervukhova *et al.* 2012; Lessing and Bonini 2008; Kayatekin *et al.* 2014). However, the proteomes of *C. albicans* and *S. cerevisiae* do not differ significantly in the proportions of Q- or N-rich proteins in their proteasomes (Figure 6). In fact, our IP-mass spec experiments identified only eight proteins that interact with 230Q in *C. albicans*, only one of which is N- or Q-rich (Table 2). Some interactions may have been missed due to technical challenges associated with aggregation-prone proteins, however, the lack of polyQ aggregation and toxicity in *C. albicans* suggests minimal functional consequences. Clearly, there is no simple correlation between the proportion of aggregation-prone proteins in the proteome of a given organism and resistance to polyQ aggregation and toxicity.

We further explored the resistance of *C. albicans* to polyQ aggregation and toxicity by directly targeting major chaperone pathways with central roles in protein quality control. We found that the chaperones Sis1 and Sgt2 interact with 230Q in *C. albicans*. These chaperones are known to assist protein folding, prevent aggregation, and target misfolded proteins for proteolytic degradation (Verghese *et al.* 2012). Sgt2 and its mammalian homolog, SGTA, are recruited into polyglutamine aggregates in *S. cerevisiae* and in mammalian cells, respectively (Wear *et al.* 2015; Wang *et al.* 2007). Sgt2 may regulate prion propagation by modulating interactions of Hsp104 and Hsp70 proteins with prion polymers (Kiktev *et al.* 2012), and may positively regulate polyQ expansions in *S. cerevisiae*. Yet the deletion of *SGT2* in *C. albicans* did not exacerbate aggregation or toxicity of polyQ expression (Figure 4, B and C). In *S. cerevisiae*, the Hsp40 chaperone Sis1 interacts with soluble polyQ species and transports polyQ aggregates, along with other misfolded proteins, to the nucleus for proteasomal degradation (Park *et al.* 2013). Depletion of *SIS1* in *S. cerevisiae* causes protein misfolding and aggregation in the cytosol (Park *et al.* 2013). Yet again, we found that deletion of *SIS1* coupled with polyQ expression had no effect on protein aggregation, solubility, or toxicity in *C. albicans* (Figure 4, B and C). Finally, even exposing *C. albicans* to geldanamycin to inhibit Hsp90 during expression of our polyQ-expanded Htt had no effect on toxicity (Figure 2C). Likewise, deletion of *HSP104*, a major fungal disaggregase, had no effect on polyQ aggregation, solubility, or toxicity (Figure 4, D and E and Figure 5).

Our results, combined with data from other model organisms, reveals a perplexing interplay between protein quality control systems, polyQ aggregation, and toxicity, and possibly more global aspects of protein misfolding. Our findings suggest that *C. albicans* has evolved powerful mechanisms to control protein aggregation during normal growth conditions and upon stress. Systematic analysis of aggregation and toxicity associated with polyQ expansion proteins, prions, and other proteins with a high propensity to misfold across different organisms and cell types will be central to elucidating fundamental mechanisms that have evolved across the tree of life to mitigate proteotoxicity.

## Supplementary Material

Supplemental material is available online at www.g3journal.org/lookup/suppl/doi:10.1534/g3.116.035675/-/DC1.
