# Supplementary material for: Candida albicans Is Resistant to Polyglutamine Aggregation and Toxicity
Source: G3 (Bethesda). 2016 Nov 1;7(1):95–108. doi: 10.1534/g3.116.035675 (PMC5217127; doi:10.1534/g3.116.035675)
Supplement: Supplementary file 1 [file 95FigureS1.docx]

**Figure S1** Expression of up to 72Q does not aggregate or cause toxicity in *C. albicans.* (.tif, 1016 KB)

Available for download as a .tif file at [www.g3journal.org/lookup/suppl/doi:10.1534/g3.116.035675/-/DC1/FigureS1.tif](http://www.g3journal.org/lookup/suppl/doi:10.1534/g3.116.035675/-/DC1/FigureS1.tif)
