# Supplementary figures and images for: Candida albicans Is Resistant to Polyglutamine Aggregation and Toxicity
Source: G3 (Bethesda). 2016 Nov 1;7(1):95–108. doi: 10.1534/g3.116.035675 (PMC5217127; doi:10.1534/g3.116.035675)

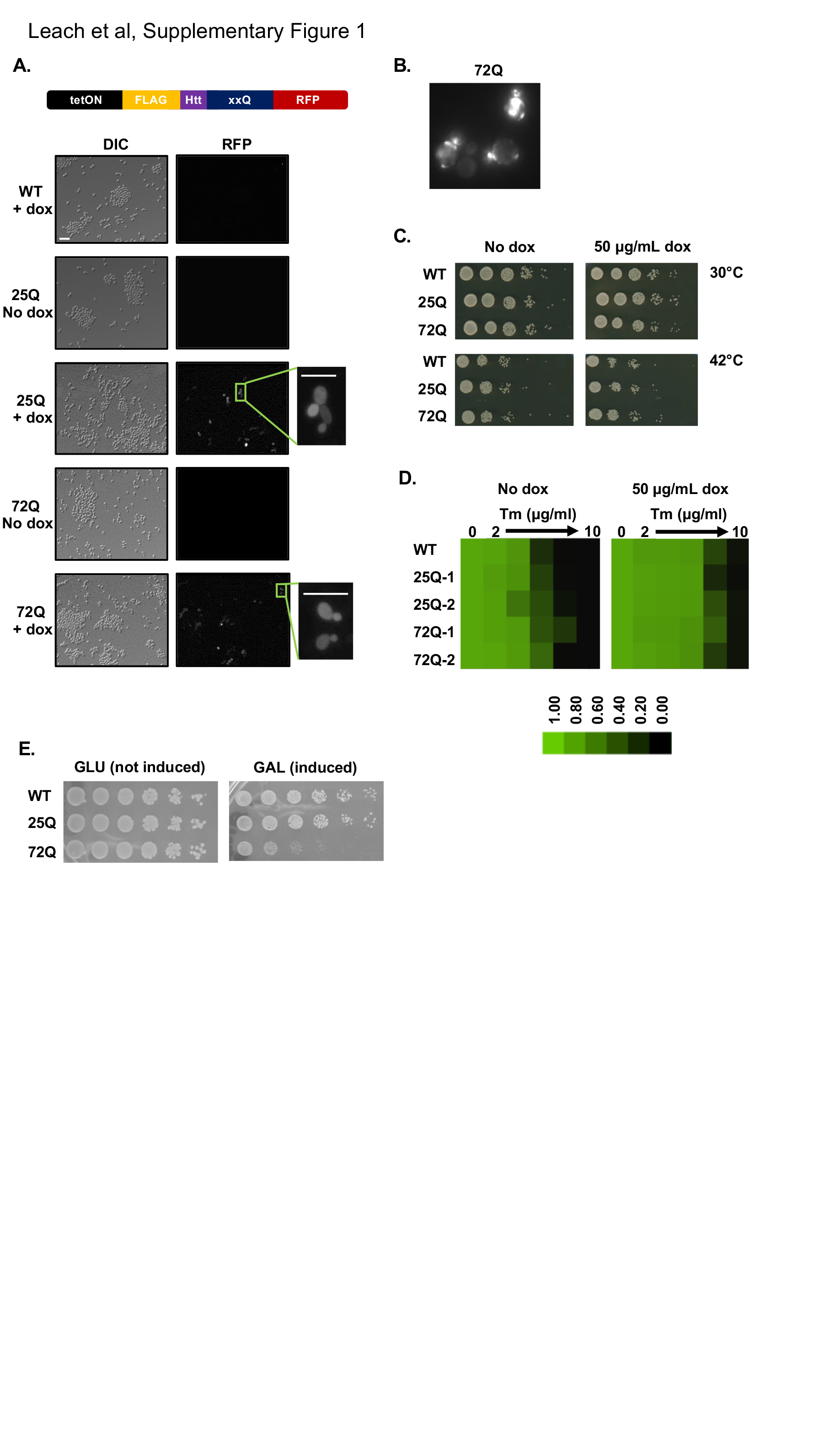

Supplement: Supplementary file 2 [file 95FigureS1.tif]

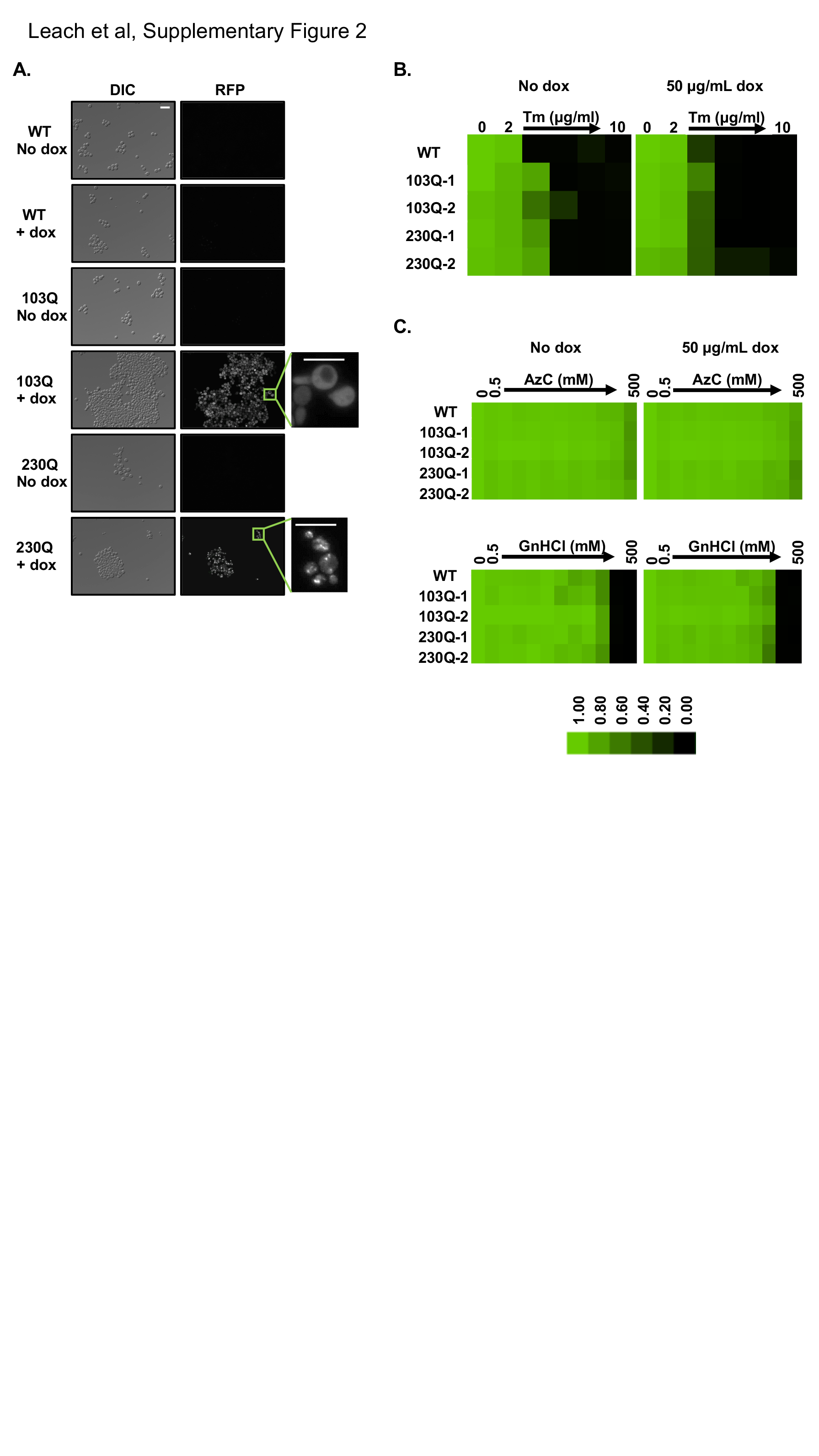

Supplement: Supplementary file 4 [file 95FigureS2.tif]

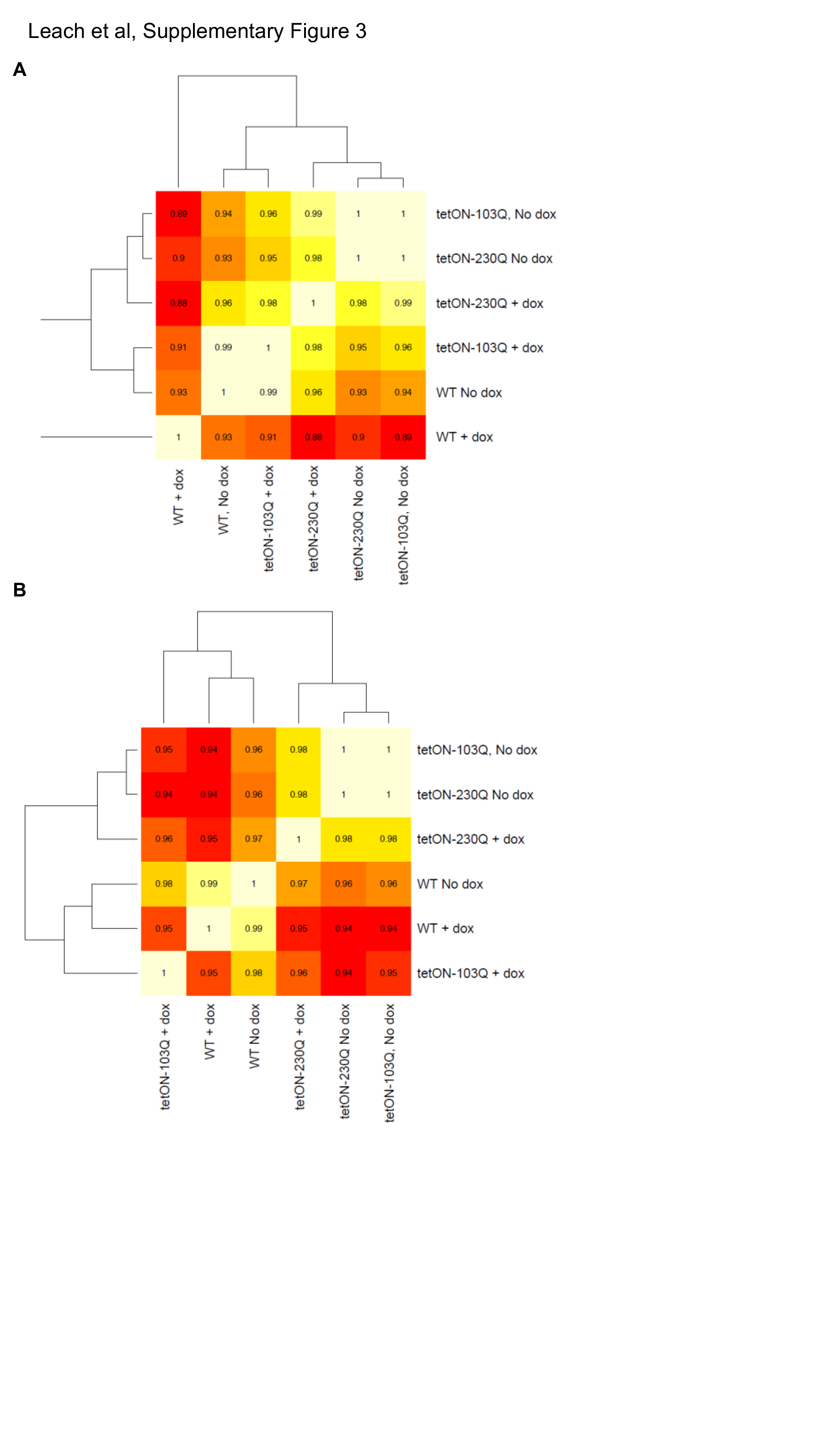

Supplement: Supplementary file 6 [file 95FigureS3.tif]
