# Supplementary material for: Candida albicans Is Resistant to Polyglutamine Aggregation and Toxicity
Source: G3 (Bethesda). 2016 Nov 1;7(1):95–108. doi: 10.1534/g3.116.035675 (PMC5217127; doi:10.1534/g3.116.035675)
Supplement: Supplementary file 5 [file 95FigureS3.docx]

**Figure S3** The *C. albicans* transcriptome is not altered upon expression of different polyQ expansions. (.tif, 548 KB)

Available for download as a .tif file at [www.g3journal.org/lookup/suppl/doi:10.1534/g3.116.035675/-/DC1/FigureS3.tif](http://www.g3journal.org/lookup/suppl/doi:10.1534/g3.116.035675/-/DC1/FigureS3.tif)
