# Supplementary material for: Candida albicans Is Resistant to Polyglutamine Aggregation and Toxicity
Source: G3 (Bethesda). 2016 Nov 1;7(1):95–108. doi: 10.1534/g3.116.035675 (PMC5217127; doi:10.1534/g3.116.035675)
Supplement: Supplementary file 7 [file 95FileS1.docx]

**File S1** RNA-sequencing of wild-type, 103Q and 230Q expressing *C. albicans* in the absence and presence of doxycycline. (.xlsx, 1097 KB)

Available for download as a .xlsx file at [www.g3journal.org/lookup/suppl/doi:10.1534/g3.116.035675/-/DC1/FileS1.xlsx](http://www.g3journal.org/lookup/suppl/doi:10.1534/g3.116.035675/-/DC1/FileS1.xlsx)
