# Supplementary material for: Candida albicans Is Resistant to Polyglutamine Aggregation and Toxicity
Source: G3 (Bethesda). 2016 Nov 1;7(1):95–108. doi: 10.1534/g3.116.035675 (PMC5217127; doi:10.1534/g3.116.035675)
Supplement: Supplementary file 9 [file 95TableS1.docx]

**Table S1** Primers (.pdf, 22 KB)

Available for download as a .pdf file at [www.g3journal.org/lookup/suppl/doi:10.1534/g3.116.035675/-/DC1/TableS1.pdf](http://www.g3journal.org/lookup/suppl/doi:10.1534/g3.116.035675/-/DC1/TableS1.pdf)
