# Supplementary material for: Candida albicans Is Resistant to Polyglutamine Aggregation and Toxicity
Source: G3 (Bethesda). 2016 Nov 1;7(1):95–108. doi: 10.1534/g3.116.035675 (PMC5217127; doi:10.1534/g3.116.035675)
Supplement: Supplementary file 10 [file 95TableS1.pdf]

Leach et al. *Candida albicans* is Resistant to Polyglutamine Aggregation and Toxicity

Supplemental Material

Table S1. Primers

Number	Gene	Sequence
oLC3001	SalI FLAG PolyQ F	TACGACTT**GTCGAC**ATGGACTACAAGGACGACGATGACAAGtTGGCGACCtTGGAAAAGtTGATGAAGGC
oLC3035	Linker-BamHI-RFP R	cttcttcaccttttgaaacGGATCCCCCGGGCTGCAG
oLC3011	BgslII RFP R	GGCAAGATCTttatttatataattcatccataccacc
oLC3036	RFP-Linker-BamHI F	CTGCAGCCCGGGGGATCCgtttcaaaaggtgaagaag
oLC3012	TetON-PolyQ F	CTCTTTCAACTTCTCAGAAAG
oLC3013	RFP-PolyQ R	CTGAACCTTCCATATGAAC
oLC3014	RFP-PolyQ F	CAATATGAAAGAGCTGAAGG
oLC3015	SAT1-PolyQ R	CAAGATATAGAAATGCCTTGG
oLC452	CaADH1-750F	TTCTCATATAGGTCATGTGC
oLC453	pNIM1 + 935R	CCATTAAGCAACTCTAATGC
oLC454	pNIM1 + 5879F	CTTGTCTGGTTACACTCACG
oLC455	CaADH1 + 2212R	TTGCTTATCAACTGGTGTCC
oLC3967	CaHSP104 F pLC49	TCATTTCTCTTTTTTTCTTTTTTTTTTCAACTTCAATTCATTATTAATAACAATTATACACTATTAGATCGGAAACAGCTATGACCATG
oLC3968	CaHSP104 R pLC49	TTATTTTTTAAAAACTATATAAAACTGTACAAAATATTAGATTTTTATTTATGTGTAATGTACTTTTAGTGTAAAACGACGGCCAG
oLC275	pJK863up-R	AAAGTCAAAGTTCCAAGGGG
oLC3021	CaHSP104 -164F	GAGTCGCAAACATTGTATG
oLC274	pJK863down-F	CTGTCAAGGAGGGTATTCTGG

oLC3969	CaHSP104d Ext R (+3166 bp)	CAGCACCAGAATATTTCTAC
oLC782	Hsp104+1409-F	CTAGTCATGAACAATTGACTGC
oLC785	Hsp104+1813-R	GTTGGTTAGGATTAGCCAAACC
oLC4197	CaSGT2-pLC49 F	AATTTTCCAAATCCATAGACAACACGTTTCCAAAACACTTTTCCCCACATCAACTAACCCATATTTCCTG**GGAAACAGCTATGACCATG**
oLC4198	CaSGT2-pLC49 R	CACAGATGAAAGAAAGCTATATAACAAATCAAACAAAAAAAAGTACTTCAATTACCCTGTTCTAATTAAA**GTAAAACGACGGCCAG**
oLC4199	CaSGT2d Ext F (-355 bp)	GCAGCACTGAATTATCAAAG
oLC4200	CaSGT2d Ext R (+1460 bp)	CACCTCCCATATTTTATACG
oLC4201	CaSGT2d Int F (+187 bp)	GAAAGTCGTTGTCAGAATTG
oLC4202	CaSGT2d Int R (+615 bp)	CGCATCTGATTTAGTTTCTC
oLC4270	CaSIS1-pLC49 F	GTTTTCATTTTCATAACCCTAAAAGAGTCAATTGATTTTAATTTAATATCAAAAATAGAATAATTGCATA**GGAAACAGCTATGACCATG**
oLC4271	CaSIS1-pLC49 R	ATTAACTAAATTACTAATAATGATATTATCGTATGCATTATTCTCTACTCCTATACTTATTATACAATAT**GTAAAACGACGGCCAG**
oLC4272	CaSIS1d Ext F (-271 bp)	CTTCTGGTGAATTCTACTTC
oLC4273	CaSIS1d Ext R (+1624 bp)	GGATGGGAGTTTTGTTAATG
oLC4378	CaSIS1 +391F	GATCACGGATTCACATATAG
oLC4379	CaSIS1 +663R	CTTTGTTCCTGATTTCCAAC
oLC1620	Hsp104+1402-F	CGAGCTAGTCATGAACAATTG
oLC1621	Hsp104+1660-R	GGATACCAGTCAATCTAGCA
oLC756	HSP90+1051-F	GCTGAAGAGTTGATTCCAGAAT
oLC757	HSP90+1236-R	GGAGAAAGCAGTGTAGAATTGG
oLC2285	CaACT1+855-F	GACCTTGAGATACCCAATTG
oLC2286	CaACT1+1076-R	CAGCTTGAATGGAAACGTAG
